# Motivated by worry, burdened by hopelessness: Emotional correlates of climate action and personal well‐being

**DOI:** 10.1111/aphw.70183

**Published:** 2026-07-01

**Authors:** Christina Saalwirth

**Affiliations:** ^1^ Department of Psychology University of the Bundeswehr Munich Neubiberg Germany

**Keywords:** climate action, climate change, concern, emotion, health

## Abstract

As public awareness of climate change grows, individuals increasingly report experiencing negative emotions such as eco‐worry and eco‐hopelessness. This study investigated how these two emotions are associated with psychological well‐being and pro‐environmental behavior (PEB) and whether the focus of these emotions (individual/micro vs. collective/macro) influences these relationships. Two cross‐sectional studies were conducted with adult participants from Germany. Study 1 included 339 individuals aged between 18 and 76 years (*M* = 33.17, *SD* = 15.76; 53.4% male, 46.0% female, 0.6% diverse), and Study 2 included 313 individuals (*M* = 34.93, *SD* = 15.70, 18–84 years; 50.2% male, 49.8% female). Analyses revealed that eco‐hopelessness was consistently associated with lower emotional well‐being and life satisfaction, whereas eco‐worry showed no associations with well‐being. In contrast, eco‐worry was robustly related to PEB, whereas eco‐hopelessness showed no association. Multigroup structural equation modeling further supported the replication of these results across both studies. In addition, the micro–macro perspectives showed distinct relations: Micro eco‐hopelessness was more strongly linked to lower well‐being, whereas macro eco‐worry was more strongly associated with PEB. These findings highlight the importance of distinguishing between specific climate‐related emotions and recognizing differential relations for an individual versus a collective focus.

## INTRODUCTION

Climate change poses not only environmental and economic challenges but also might have profound psychological consequences. Individuals increasingly report negative emotional reactions—such as worry, anxiety, and hopelessness—in response to the climate crisis (Konrad‐Adenauer‐Stiftung, 2023; Marlon et al., 2022). Although such emotions reflect a growing awareness of the severity of climate change, their implications for psychological well‐being and pro‐environmental behavior (PEB) remain complex and multifaceted. For example, research has shown that eco‐worry can yield both beneficial and harmful outcomes (Ojala et al., 2021). On the one hand, it may serve as a motivator for engaging in PEB (Bouman et al., 2020); on the other hand, eco‐worry may lead to increased emotional distress, including a heightened risk of depression (Sciberras & Fernando, 2022). However, the link between eco‐worries and well‐being remains inconclusive as not all studies support this association (e.g. Verplanken & Roy, 2013). Worry can be accompanied by feelings of hopelessness when negative events are appraised as certain, and no coping mechanisms are available to prevent harmful outcomes. Hopelessness has been consistently associated with negative mental health outcomes and disengagement from action (Liu et al., 2015; Marchetti et al., 2023), yet eco‐hopelessness in particular remains underexplored in the climate change literature. In addition, the distinct and combined associations of eco‐worries and eco‐hopelessness with well‐being and PEB warrant further empirical investigation. Especially the differentiation between an individual (micro) and a collective (macro) perspective on worry described by Boehnke et al. (1998), but also on hopelessness, has received little attention in environmental psychology.

The two studies presented, therefore, aim to clarify the differential roles of eco‐worry and eco‐hopelessness in relation to well‐being and PEB and further examine whether the object of eco‐worry and eco‐hopelessness—namely, a micro or a macro perspective—differs in their relation to well‐being and PEB.

### Eco‐worry

Worry is one of many concepts investigated over the past decades to describe the negative emotions and cognitions that may arise when dealing with the climate crisis (Pihkala, 2022). Worry encompasses emotional and cognitive elements, with the latter playing a dominant role (Borkovec et al., 1998). Worrying is characterized by uncontrollable repetitive thoughts and mental images about possible future threats, often accompanied by negative emotions, primarily fear and anxiety (Andrews & Borkovec, 1988; Borkovec et al., 1983). In the appraisal theory of emotion (Smith & Kirby, 2011; Smith & Lazarus, 1990), worry can be understood as an emotion that emerges from a particular pattern of cognitive appraisals—specifically, within situations are appraised as stressful. According to this theory, emotions arise from the combination of primary and secondary appraisals. Primary appraisals evaluate the relevance of a situation to a person's well‐being and whether it is congruent or incongruent with personal goals (i.e. desirable or undesirable). Secondary appraisals assess coping potential (i.e. manageable or unmanageable). In accordance with this theory, worrying is the emotional result of appraising a situation as important and potentially harmful (stressful), with uncertain perceived ability to cope or adjust to it.

Eco‐worry specifically describes the persistent and intrusive thoughts and images related to the anticipated consequences of environmental degradation and the climate crisis (Ojala et al., 2021). In line with the appraisal theory of emotion, it was classified as a threat‐related climate emotion by Pihkala (2022) in her taxonomy of environmentally relevant emotions and mental states and reflects a specific form of future‐oriented stress appraisal (Lazarus & Folkman, 1984; Saalwirth & Leipold, 2023). This appraisal arises in response to perceived threats to ecological systems, one's personal well‐being, or the well‐being of future generations. Therefore, eco‐worry is distinct in its focus on environmental issues and is increasingly recognized as a significant psychological response to the growing awareness of the psychological consequences of climate change (Ojala et al., 2021).

Although the concept of eco‐worry is clearly defined, it is often studied within the broader context of eco‐anxiety (see, e.g. Lutz et al., 2023 and Verplanken et al., 2020). Eco‐anxiety represents a construct that includes various stress‐related responses to climate change, such as fear, concern, functional impairment, and depression (Clayton & Karazsia, 2020). Similarly, Kurth and Pihkala (2022) describe eco‐anxiety as a family of distinct but related emotions. While eco‐anxiety encompasses a wide range of symptoms, worry represents one of these components (Ágoston et al., 2022; Verplanken et al., 2020). Nonetheless, I argue that it is essential to differentiate between these two concepts. Eco‐anxiety encompasses a broad range of psychological responses, often emphasizing clinically relevant symptoms (e.g. functional impairment, distress, or depressive features). In contrast, eco‐worry is a narrowly defined construct that does not inherently imply clinical relevance (Zebb & Beck, 1998). Although eco‐worry may involve persistent concern about environmental issues, it can also reflect a normative and potentially adaptive response to the climate crisis, rather than a sign of psychopathology (Verplanken & Roy, 2013). Moreover, worry and anxiety are also clearly distinguishable within the appraisal theory of emotion (Smith & Kirby, 2011; Smith & Lazarus, 1990). Worry is primarily a cognitive process involving repetitive thoughts about potential future threats. It arises when individuals appraise a situation as important and undesirable, but still believe that some degree of coping is possible. In this sense, worry involves a sense of uncertainty and concern, yet is still marked by a readiness to take action. In contrast, anxiety is primarily an emotional state characterized by feelings of tension, arousal, and vulnerability. It emerges when coping appraisals are perceived as low, and the individual doubts their ability to adapt to the anticipated threat. While worry tends to be verbal, deliberate, and future‐oriented, anxiety is characterized by immediate physiological arousal and symptoms such as restlessness or a racing heart.

#### Relations with well‐being

Research on the relationship between eco‐worry and well‐being has yielded mixed findings. While some studies suggest that eco‐worry is linked to lower well‐being, others do not find such an association. For instance, Wullenkord and Ojala (2023) reported that climate change worry was positively associated with negative affect and negatively associated with life satisfaction, though the latter correlation was weak. Similarly, Sciberras and Fernando (2022) found that adolescents with persistently high levels of eco‐worry exhibited higher rates of depression in a longitudinal study. McBride et al. (2021) also observed that climate concern predicted a slight increase in psychological distress, including symptoms of depression and anxiety. However, their study did not find a connection between climate concern and life satisfaction. Likewise, Verplanken and Roy (2013) found no association between habitual eco‐worry and pathological worry.

A distinction between micro and macro worry may help account for these inconsistencies. This differentiation was introduced by Boehnke et al. (1998) and describes two separate objects of worry: Micro worry focuses on oneself and close others, whereas macro worry focuses on society or the world in general. Macro worry, therefore, is not intended to capture highly specific or personally concrete concerns but rather reflects a more generalized, societal‐level orientation of worry. This differentiation turned out to be especially relevant when exploring the relationship between worry and well‐being because only micro worry was consistently negatively associated with impairments of well‐being. Macro worry showed no or small positive associations with mental health (Boehnke et al., 1998; Schwartz & Melech, 2000). To date, only limited empirical work has examined these two forms of worry in the context of climate change. Wullenkord and Ojala (2023) found that, consistent with general findings, micro eco‐worry is more closely associated with lower well‐being than macro eco‐worry. However, these findings have yet to be replicated.

#### Relations with PEB

A growing body of research suggests that eco‐worry can serve as a motivating force for PEB. For instance, Bouman et al. (2020) found that individuals who experienced greater levels of worry about climate change were more likely to engage in and support climate‐related actions. Similarly, Stevenson and Peterson (2016) reported a positive relationship between worry about climate change and actual climate action. Verplanken et al. (2020) further demonstrated that eco‐worry is associated with a broader pro‐ecological worldview, strong pro‐environmental values, and a history of environmentally responsible behavior. In addition, Verplanken and Roy (2013) found that individuals who reported habitual eco‐worry tended to hold more pro‐environmental attitudes and were more likely to engage in eco‐friendly behaviors. Collectively, these findings highlight eco‐worry as a potentially adaptive emotional response that can motivate environmental engagement. Further, Wullenkord and Ojala (2023) found that, in relation to PEB, macro eco‐worry demonstrated a stronger association with PEB than micro eco‐worry. As the authors discuss, these results may be explained by differences in underlying value orientations. Wullenkord and Ojala (2023) argue that macro worries might reflect biospheric and altruistic concern, previously described by Schultz (2001), which has been found to predict PEB. In contrast, micro worries might resemble egoistic concern, which usually predicts less engagement in PEBs. However, like the distinction in micro and macro eco‐worries and their relations to well‐being, these findings need replication.

### Eco‐hopelessness

Similar to eco‐worry, eco‐hopelessness also describes a negative emotional response to climate change. In general, hopelessness is defined by three central components: the belief that meaningful and desired outcomes are unlikely to occur (negative expectations), the belief that one cannot influence or alter these outcomes (a sense of helplessness), and a blocked goal‐pursuit (Abramson et al., 1989; Marchetti et al., 2023). Within the appraisal theory of emotion (Smith & Kirby, 2011; Smith & Lazarus, 1990), hopelessness arises when individuals appraise a situation as both highly significant and severely misaligned with their goals, while simultaneously perceiving no viable means of change or adaptation. Therefore, hopelessness reflects a combination of high motivational relevance, high motivational incongruence, and low appraisals of coping potential. Critically, it is also characterized by low future expectancy—the belief that the situation is unlikely to improve.

Eco‐hopelessness, in particular, refers to outcomes related to climate change, such as loss of species, water scarcity, rising temperatures, or refugee movements. Eco‐hopelessness can be understood as a stress appraisal process in which individuals evaluate potential future environmental threats as certain, overwhelming, and beyond their capacity to manage (Lazarus & Folkman, 1984). This response emerges when a person perceives the challenges posed by climate change as insurmountable and concludes that personal or collective resources, such as knowledge, coping strategies, or willingness to change, are inadequate to effectively address the problem of climate change. As a result, eco‐hopelessness reflects a sense of despair and powerlessness in the face of environmental degradation that may negatively impact the well‐being of individuals and may lead to emotional disengagement, reduced motivation, and a diminished sense of agency.

#### Relations with well‐being

Previous research has shown that hopelessness, in general, is associated with depression and suicidality (Liu et al., 2015; Marchetti et al., 2023), which is why it was also classified as a climate change feeling strongly related to depression by Pihkala (2022). Although there is evidence that hopelessness can significantly impair an individual's well‐being (Çapri et al., 2013; Satici & Uysal, 2017), research specifically addressing eco‐hopelessness remains limited. Although considerable attention has been given to the role of hope in the context of climate change and well‐being (e.g. Geiger et al., 2021; Ojala, 2012; Stevenson & Peterson, 2016), hopelessness has received far less empirical focus in environmental psychology. However, as Huen et al. (2015) argue, hope and hopelessness are not simply two sides of the same coin; instead, they are correlated but conceptually distinct constructs. Requero et al. (2021) further emphasize that these constructs reflect different ways of appraisal: Hope is appraised as a positive emotional state characterized by uncertainty, whereas hopelessness is appraised as a negative emotional state marked by a perceived certainty of adverse outcomes. Given the growing emotional toll of the climate crisis, this lack of research into the implications of eco‐hopelessness represents a critical gap in understanding how negative emotional responses to environmental threats may uniquely impact well‐being.

Furthermore, applying the micro–macro distinction used in eco‐worry research to eco‐hopelessness could yield valuable insights and reveal unique patterns in its association with well‐being, an area that remains unexplored in current research. In the context of eco‐hopelessness, a micro perspective involves a pessimistic outlook on one's own future in relation to climate threats. In contrast, a macro perspective reflects negative expectations for society or the world. Recognizing these different objects of eco‐hopelessness may be crucial, as they could differentially influence psychological well‐being.

#### Relations with PEB

Previous research has shown that hopelessness is often linked to passivity and disengagement, which is in line with the appraisal theory of emotion (Smith & Kirby, 2011; Smith & Lazarus, 1990). Marchetti et al. (2023) even consider passivity a key component of hopelessness (blocked goal‐pursuit). Therefore, one would expect eco‐hopelessness to be associated with less PEB. However, empirical data to support this assumption are lacking. Similar to relations with well‐being, research focused on investigating hope rather than hopelessness, which is positively associated with PEB when constructive (Marlon et al., 2019; Ojala, 2012). Nevertheless, helplessness—a core element of hopelessness—has been shown to be negatively associated with PEB (Salomon et al., 2017), supporting the idea that eco‐hopelessness might also be linked to lower levels of PEB. Whether this holds true and whether differences exist between micro and macro eco‐hopelessness need further investigation.

## STUDY 1

The first of the two studies presented aims to examine the distinct roles of eco‐worry and eco‐hopelessness in relation to psychological well‐being and PEB. While eco‐worry has received increasing attention in environmental psychology, eco‐hopelessness remains a largely underexplored construct. Given its potential influence on psychological well‐being and environmental engagement, a more in‐depth exploration of eco‐hopelessness is essential. This study seeks to fill this gap by exploring how these two emotional responses to climate change may differentially be related to individuals' well‐being and their willingness to act in environmentally friendly ways with structural equation models (SEMs). Previous research has shown that worry is related to hopelessness (Beck et al., 2001). Therefore, eco‐worry and eco‐hopelessness may share overlapping associations with well‐being, suggesting that accounting for one could alter the observed relationship of the other.

Due to consistent findings that link hopelessness with lower well‐being (e.g. Satici & Uysal, 2017) and the inconclusive relationship between eco‐worry and well‐being, I suspect eco‐hopelessness to be more strongly associated with well‐being than eco‐worry. This assumption is also in line with the appraisal theory of emotion (Smith & Kirby, 2011; Smith & Lazarus, 1990) as hopelessness involves the belief that one lacks both the ability to successfully cope and any expectation that the situation will improve. In contrast, eco‐worry is marked by uncertainty about future outcomes and one's coping ability, which may sustain concern but does not imply a complete loss of control. I further hypothesize, as previous research has shown, that while eco‐worry will be positively related to PEB (e.g. Bouman et al., 2020; Stevenson & Peterson, 2016), eco‐hopelessness may be associated with lower levels of PEB, given its key component of blocked goal‐pursuit (Marchetti et al., 2023). This again is supported by the appraisal theory of emotion (Smith & Kirby, 2011; Smith & Lazarus, 1990). Whereas eco‐worry reflects a state of concern that retains some perceived capacity to act, and therefore might motivate PEB, eco‐hopelessness involves the appraisal that no effective action is possible, likely reducing the motivation to engage in goal‐directed environmental action. Therefore, the following hypotheses were formulated:

1. Eco‐hopelessness and eco‐worry are negatively related to well‐being.
  - Eco‐hopelessness shows a stronger association with well‐being than eco‐worry.
2. Eco‐worry is positively related to PEB.
3. Eco‐hopelessness is negatively related to PEB.

### Method

An online questionnaire collected descriptive data on eco‐worry, eco‐hopelessness, well‐being, and PEB. The questionnaire was distributed via mailing lists and social media in Germany. No monetary compensation was given to the participants, and all participants gave informed consent. The study was approved by the institutional Ethics Committee of the University of the Bundeswehr Munich. The study was not preregistered.

#### Study sample

Data on 360 individuals were collected. The only inclusion criterion was a minimum age of 18 years. All participants provided informed consent. Of these 360 participants, 6 were excluded due to missing data, 6 due to being underaged, and 9 due to conspicuous response patterns, resulting in a final sample of *N* = 339 participants aged between 18 and 76 years (*M* = 33.17, *SD* = 15.76). The gender distribution was 53.39% male and 46.02% female, with two participants identifying as diverse. A university degree was reported by 38.34% of the sample. Regarding household income, 16.81% of the participants earned less than €1000 per month (low‐income earners), 55.16% earned up to €3000 a month, and 28.01% earned more than €3000 a month.

#### Instruments

Apart from the descriptive statistics, eco‐worry, eco‐hopelessness, well‐being, and PEB were measured with the questionnaires described in further detail below. Items originally in English were first translated into German using an AI‐based translation tool (DeepL). The translations were then reviewed and double‐checked for accuracy, clarity, and cultural appropriateness by the author and two independent colleagues.

The Climate Change Worry Scale (CCWS; Stewart, 2021) was used to measure eco‐worry. This scale assesses proximal worries about climate change with 10 items. The scale provides good reliability and validity (Stewart, 2021). Participants indicate how frequently each of the 10 statements applies to them on a 5‐point Likert scale, where higher scores reflect more frequent eco‐worries. A mean score was calculated. Reliability for the data was good (*α* = .92).

Eco‐hopelessness was assessed using four adapted items of Beck's Hopelessness Scale (Beck et al., 1974), where several authors supported a single‐factor solution (e.g. Aish & Wasserman, 2001). The items can be found in Table S1 (macro eco‐hopelessness). The scale ranged from 1 to 7, with higher scores indicating a greater feeling of eco‐hopelessness. A confirmatory factor analysis (CFA) was conducted to examine the factor structure of the eco‐hopelessness scale using a one‐factor model. The model showed an acceptable fit to the data: *χ* ^2^(2) = 30.45, *p* < .001; comparative fit index (CFI) = .94; Tucker–Lewis index (TLI) = .81; root mean square error of approximation (RMSEA) = .20; standardized root mean square residual (SRMR) = .04. All factor loadings were significant (*p* < .001), standardized estimates ranging from .42 to .82, indicating that all items contributed meaningfully to the latent construct. Reliability was good (*α* = .78).

Well‐being was assessed through emotional well‐being and life satisfaction. Emotional well‐being was assessed using the 5‐item World Health Organization Well‐Being Index (WHO‐5). Participants rated their emotional well‐being over the past 4 weeks on a 6‐point Likert scale, with higher scores reflecting greater well‐being. Reliability was good (*α* = .81). Life satisfaction was evaluated with the Satisfaction With Life Scale (SWLS; Diener et al., 1985), comprising five items with a 7‐point Likert scale. Higher scores indicate higher life satisfaction. Reliability was good (*α* = .86).

PEB was assessed using five self‐developed items designed to capture a range of individual climate‐related actions. Participants responded on a 5‐point Likert scale ranging from 1 (*never*) to 5 (*nearly all the time*), with higher scores indicating greater engagement in PEB. Items reflected various aspects of sustainable behavior, including reducing climate‐damaging activities, making personal sacrifices for the environment, and encouraging others to act sustainably. A mean score was calculated to represent overall PEB. The complete list of items can be found in Table S2. To assess the factorial structure of the scale, a CFA was conducted testing a one‐factor model. Model fit indices indicated that the one‐factor solution provided an excellent fit to the data: *χ* ^2^(5) = 9.40, *p* = .09; CFI = .99; TLI = .99; RMSEA = .05; SRMR = .02. All factor loadings were statistically significant (*p* < .001), with standardized estimates ranging from .66 to .79. These results support the unidimensionality of the PEB construct. The scale demonstrated good internal consistency (*α* = .84).

#### Statistical analysis

The relationships among eco‐worry, eco‐hopelessness, well‐being, and PEB were examined using a stepwise modeling strategy involving three SEMs with increasing constraints, applying a significance threshold of *p* < .05. The precise specification of the structural models was informed by both theoretical reasoning and empirical observation.

In the first step, the latent covariance baseline model (M1) allowed all latent constructs to freely covary and served as a baseline. This model approximates the observed correlation matrix without imposing directional or structural assumptions while controlling for age and gender. The control variables age and gender were defined as predictors for emotional well‐being, life satisfaction, PEB, eco‐worry, and eco‐hopelessness and were allowed to covary with each other. In the second step, the restricted covariance model (M2) retained only the theoretically and statistically relevant covariances and served as a more parsimonious comparison model to assess whether the pattern of associations could be captured with fewer estimated parameters while maintaining acceptable model fit. Therefore, it served as a transitional step between the latent covariance baseline model (M1) and the structural path model (M3). In the third step, the structural path model (M3) represented the primary hypothesis‐testing model. This model specified theory‐driven directional paths. Comparing these models provides added value by testing theoretically grounded structures, controlling for control variables (age and gender), identifying unique contributions of predictors, and controlling for shared variance among latent constructs.

The SEM analyses employed maximum likelihood (ML) estimation, with all constructs (except the control variables) modeled as latent variables indicated by their respective items. Full information ML (FIML) was used. All calculations were conducted using IBM SPSS Statistics (version 29.0.1.0) and JASP (version 0.19.1.0). There were no missing data for the variables investigated.

### Results

Descriptive statistics and bivariate correlations for all variables of Study 1 are presented in Table 1. The correlations offer a preliminary overview of the associations among eco‐worry, eco‐hopelessness, well‐being, and PEB but are not interpreted, as all main hypotheses were tested using the SEMs described below. Data and analysis materials are available online (https://osf.io/xhv4b/overview?view_only=b0940e727b07487ca0f28af470dee677).

**TABLE 1:** Descriptive statistics and correlations of Study 1.

	*M* (*SD*)	Range	1	2	3	4	5	6
1. Eco‐worry	2.57 (0.86)	1.00–5.00						
2. Eco‐hopelessness	3.21 (1.33)	1.00–7.00	.28***					
3. Emotional well‐being	3.85 (0.89)	1.60–6.00	−.00	−.26***				
4. Life satisfaction	5.15 (1.07)	1.00–7.00	.07	−.20***	.53***			
5. PEB	3.06 (0.78)	1.20–5.00	.63***	.04	.04	.03		
6. Age	33.17 (15.76)	18.00–76.00	.12*	−.02	−.01	−.08	.24***	
7. Gender	1.46 (0.50)	—	.32***	.09	−.05	.00	.27***	.09

*Note*: Gender: 1 = male, 2 = female, two participants reported diverse and were not included in the correlation with the gender variable.

Abbreviations: PEB, pro‐environmental behavior; *SD*, standard deviation.

* *p* < .05,

** *p* < .01, and

*** *p* < .001.

#### SEMs

To examine the distinct associations between eco‐worry, eco‐hopelessness, well‐being, and PEB, three SEMs with increasing theoretical constraints were estimated. Model fit and comparative statistics for all three models are reported below.

##### Latent covariance baseline model (M1)

The latent covariance baseline model (M1) allowed all latent variables—eco‐worry, eco‐hopelessness, emotional well‐being (WHO‐5), life satisfaction (SWLS), and PEB—to freely covary, while controlling for age and gender. This model fit the data well (*χ* ^2^[415] = 824.95, *p* < .001, CFI = .92, RMSEA = .05, SRMR = .06; goodness‐of‐fit index [GFI] = .98). See Table 2 for the standardized associations between the latent variables. While controlling for age and gender, shared variances of the latent constructs, and measurement error, eco‐hopelessness was negatively associated with emotional well‐being and life satisfaction, whereas eco‐worry was not related to well‐being. This supports Hypothesis 1a that eco‐hopelessness shows a stronger negative association with well‐being than eco‐worry. Notably, a reversed pattern was visible for PEB. Whereas eco‐worry was positively correlated with PEB, which supports Hypothesis 2, eco‐hopelessness was not associated with greater PEB, which contradicts Hypothesis 3.

**TABLE 2:** Standardized associations of the latent covariance baseline model (M1) and restricted covariance model (M2) of Study 1.

Association	M1	M2
Emotional well‐being ~~ eco‐hopelessness	−.30*** (.06)	−.30*** (.06)
Life satisfaction ~~ eco‐hopelessness	−.23*** (.06)	−.23*** (.06)
Emotional well‐being ~~ eco‐worry	.00 (.05)	
Life satisfaction ~~ eco‐worry	.07 (.05)	
PEB ~~ emotional well‐being	.06 (.06)	
PEB ~~ life satisfaction	.06 (.06)	
PEB ~~ eco‐hopelessness	.02 (.05)	
PEB ~~ eco‐worry	.65*** (.04)	.65*** (.04)
Life satisfaction ~~ emotional well‐being	.67*** (.04)	.67*** (.04)
Eco‐hopelessness ~~ eco‐worry	.27*** (.05)	.27*** (.05)
Gender → eco‐worry	.32*** (.05)	.30*** (.05)
Gender → PEB	.28*** (.05)	.29*** (.05)
Age → PEB	.11* (.05)	.17*** (.05)

*Note*: Covariances represent standardized associations between latent constructs. Values are shown as standardized coefficients (standard error). Age and gender were modeled as predictors of all latent variables in both models. Only theoretically and statistically relevant paths are shown for clarity; gender was coded as 1 = male, 2 = female.

Abbreviation: PEB, pro‐environmental behavior.

* *p* < .05,

** *p* < .01, and

*** *p* < .001.

Interestingly, although not one of the main research interests, there was no association between either of the two well‐being indicators and PEB, suggesting that behaving in an environmentally friendly way is not linked to better emotional well‐being or greater life satisfaction. In addition, as expected, eco‐hopelessness and eco‐worry revealed a positive correlation. Individuals who experience higher levels of eco‐worry are also more likely to feel a sense of hopelessness regarding climate change. Furthermore, a positive association was observed between emotional well‐being and life satisfaction, reinforcing the internal consistency of the well‐being measures.

##### Restricted covariance model (M2)

The restricted covariance model (M2) retained only theoretically and statistically supported covariances, excluding non‐significant associations observed in the latent covariance baseline model (M1). Specifically, the covariances between eco‐hopelessness and PEB, as well as eco‐worry and both well‐being indicators, were removed. In addition, the covariances of PEB with both well‐being indicators were excluded. Model fit remained good (*χ* ^2^[420] = 830.36, *p* < .001, CFI = .92, RMSEA = .05, SRMR = .06, GFI = .98). See Table 2 for the standardized associations between the latent variables. Compared with M1 (AIC = 30142.32; BIC = 30570.83), M2 achieved slightly lower information criteria (AIC = 30137.73; BIC = 30547.11), despite using fewer parameters (107 vs. 112). This suggests that the restricted covariance model (M2) provides a more parsimonious representation of the data. All significant associations of M1 that are included in M2 remained significant.

##### Structural path model (M3)

**FIGURE 1:**
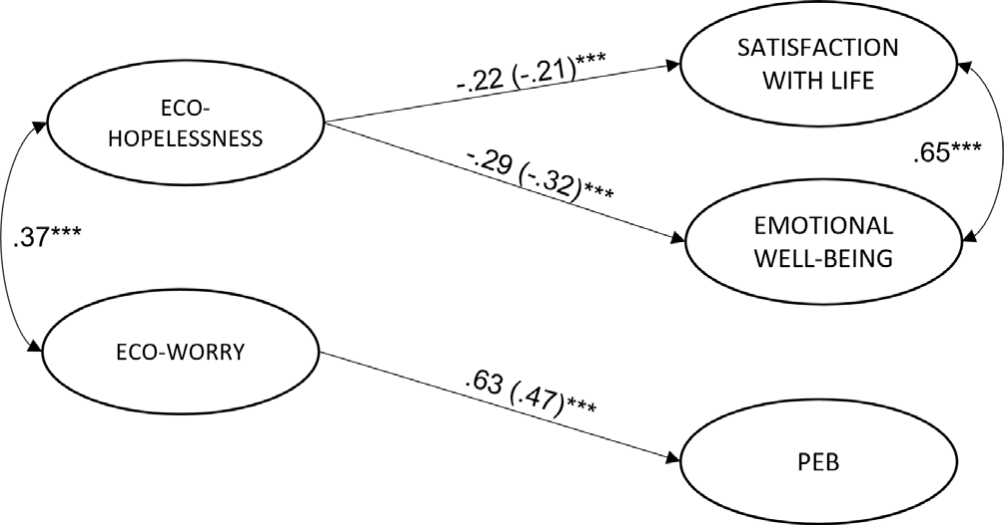
Structural path model (M3) of Study 1 and showing the prediction of well‐being (emotional well‐being and life satisfaction) by eco‐hopelessness and the prediction of PEB by eco‐worries. Paths for control variables (age, gender) are omitted for clarity. PEB, pro‐environmental behavior. Standardized regression path coefficients from Study 2 (derived from the multigroup SEM) are presented in brackets. *p < .05, **p < .01, and ***p < .001.

The structural path model (M3) further tested the hypotheses by additionally specifying directional paths from eco‐worry to PEB, and from eco‐hopelessness to emotional well‐being and life satisfaction. This structure reflects the theoretical proposition that eco‐worry functions as a motivational factor for engagement, whereas eco‐hopelessness primarily impairs psychological well‐being. Model fit remained good and comparable with the less constrained models (*χ* ^2^[420] = 843.02, *p* < .001, CFI = .92, RMSEA = .05, SRMR = .07, GFI = .98). The structural path model (M3) is depicted in Figure 1. Given its strong model fit and its ability to test directional paths, M3 is considered the most theoretically meaningful and informative of the three models. In M3, eco‐hopelessness negatively predicted emotional well‐being and life satisfaction, indicating that higher levels of eco‐hopelessness are associated with lower levels of well‐being across different well‐being domains, supporting Hypothesis 1a. In line with Hypothesis 2, eco‐worry was a strong positive predictor of PEB. As paths from eco‐worry to well‐being and from eco‐hopelessness to PEB were omitted based on the results of M1 while the overall model demonstrated good fit to the data, Hypothesis 1 stating that eco‐worry is negatively related to well‐being and Hypothesis 3 stating that eco‐hopelessness is negatively related to PEB had to be rejected.

### Discussion

Taken together, the results indicate a moderate positive association between eco‐worry and eco‐hopelessness. Individuals who are worried about environmental issues may also feel hopeless about the outcome of climate change in the future, which is in line with previous research (Beck et al., 2001). However, although related, eco‐worry and eco‐hopelessness also have distinct relationships with indicators of well‐being and PEB.

The results of Study 1 largely support Hypothesis 1, indicating that eco‐hopelessness is negatively associated with well‐being and exhibits a stronger relationship than eco‐worry. However, contrary to expectations, eco‐worry showed no meaningful association with well‐being, suggesting that general environmental worry may not be directly linked to individuals' subjective well‐being. Therefore, eco‐hopelessness appears particularly relevant when considering an individual's well‐being. This highlights that eco‐worry per se does not seem to be linked to impairments of individuals' psychological health; however, when worries may conclude in a sense of hopelessness, there might be a cost of perceiving environmental issues as unmanageable. Considering the associations with PEB, one could argue that a certain level of eco‐worry might even have positive effects, as it is strongly associated with more environmentally friendly behavior (PEB). This supports Hypothesis 3 and previous research suggesting that worries might motivate action (Bouman et al., 2020). On the other hand, in contrast to Hypothesis 3, eco‐hopelessness was not associated with PEB, suggesting that individuals who expect negative outcomes and feel a sense of helplessness regarding environmental issues are not less likely to take proactive steps.

## STUDY 2

The second study first aims to replicate the findings of Study 1, which revealed a negative relationship between eco‐hopelessness and well‐being and a positive relationship between eco‐worry and PEB. Beyond replication, Study 2 further seeks to expand on those results by examining whether these relationships differ depending on the object (micro versus macro) of eco‐worry and eco‐hopelessness. Based on previous research by Boehnke et al. (1998), I suspect micro eco‐hopelessness to be closer linked to well‐being than macro eco‐hopelessness. Further, macro eco‐worry might be closer related to PEB than micro eco‐worry, given the underlying value orientations of macro (biospheric/altruistic) and micro (egoistic) concerns (Wullenkord & Ojala, 2023). Therefore, the following hypotheses were formulated:

1. Eco‐hopelessness is negatively related to well‐being.
  - This relationship is stronger for micro than for macro eco‐hopelessness.
2. Eco‐worry is not related to well‐being.
3. Eco‐worry is positively related to PEB.
  - This relationship is stronger for macro than for micro eco‐worries.
4. Eco‐hopelessness is not related to PEB.

### Method

The data on eco‐worry (micro and macro), eco‐hopelessness (micro and macro), well‐being, and PEB were gathered through an online questionnaire, which was distributed via mailing lists and social media in Germany. No monetary compensation was given to the participants, and all participants gave informed consent. The study was approved by the institutional Ethics Committee of the University of the Bundeswehr Munich. The study was not preregistered.

#### Study sample

A total of 316 participants completed the questionnaire, of whom three had to be deleted due to missing data or conspicuous response patterns. The final sample consisted of *N* = 313 participants between 18 and 84 years old (*M* = 34.93, *SD* = 15.70). The gender ratio was 50.2% men to 49.8% women. Regarding educational background, 41.2% of participants reported having completed higher school education (Fachhochschulreife or Abitur), 20.8% held a university degree at the bachelor's level or higher, and 17.4% had lower secondary education or no qualification. In terms of household net income, 13.4% of participants reported earning €0–€1000, 17.6% earned €1001–€2000, and 46.3% earned €2001–€3000 per month. Higher income was reported by 22.7% (>€3001) of the participants. One participant (0.3%) chose not to report their income. The only inclusion criterion was a minimum age of 18 years. Participants gave informed consent.

#### Instruments

Eco‐worry, eco‐hopelessness, well‐being, and PEB were measured with the questionnaires described in further detail below. Items originally in English were first translated into German using an AI‐based translation tool (DeepL). The translations were then reviewed and double‐checked for accuracy, clarity, and cultural appropriateness by the author and two independent colleagues.

The same as in Study 1, eco‐worry was assessed with the CCWS (Stewart, 2021). Reliability for the data was good (*α* = .92). In addition, to assess micro (individual) and macro (collective) eco‐worry, the measure from Schultz (2001), which assesses environmental worry from both perspectives, was used. Each type of worry was measured using five items (see Table S3), asking participants to rate their level of eco‐worry on a scale from 1 to 5, where higher scores reflect greater eco‐worries. Mean scores for both micro and macro eco‐worries were calculated. To examine the factorial structure of the scale, a CFA specifying two correlated latent factors representing micro and macro eco‐worries was conducted. Modification indices revealed that Item 5 of each subscale showed evidence of cross‐loading and shared residual variance with items from the other factor. Conceptually, this may be due to the differing objects of worry in these items: The macro Item 5 referred to harm to nature and animals, in contrast to society, whereas the micro item referred to harm to one's family in contrast to oneself. These thematic inconsistencies may have undermined the distinction between the two factors. After removing these two items, the model showed excellent fit (CFI = .99, TLI = .98, RMSEA = .06, SRMR = .02). Standardized factor loadings ranged from .58 to .90 for micro eco‐worry and from .81 to .92 for macro eco‐worry and were all statistically significant. The resulting four‐item subscales demonstrated good reliability (*α*_Micro_ = .86; *α*_Macro_ = .92). Cronbach's alpha remained stable if items were dropped (*α*_Micro_ > .79; *α*_Macro_ > .88), and item‐rest correlations were within the acceptable range (*r*_*ir(Micro)*_ > .55; *r*_*ir(Micro)*_ > .78; the exact statistics can be found in Table S3). Average variance extracted (AVE) of the two latent factors explained more than 50% of their indicators' variance (AVE_Micro_ = .64, AVE_Macro_ = .75) indicating good convergent validity. In addition, high correlations of the micro (*r* = .77, *p* < .001) and the macro (*r* = .77, *p* < .001) dimension with the CCWS further supports good convergent validity. Discriminant validity was assessed using the heterotrait–monotrait (HTMT) ratio. The HTMT value between *micro and macro eco‐worries* was .69, supporting discriminant validity (Henseler et al., 2015).

Macro (collective) eco‐hopelessness was assessed using the same four adapted items from the Beck Hopelessness Scale (Beck et al., 1974) as in Study 1. Micro (individual) eco‐hopelessness was measured with the same items, but rephrased to reflect a personal or individual perspective. All items can be found in Table S1. Responses were recorded on a 7‐point scale, with higher scores indicating greater levels of eco‐hopelessness. To examine the factorial structure of the scales, I conducted a CFA specifying two correlated latent factors representing micro and macro eco‐hopelessness. The two‐factor model showed substantially better fit than a one‐factor model: CFI = .95 versus .83, TLI = .91 versus .70, RMSEA = .12 versus .23, and SRMR = .05 versus .08, supporting the distinction between micro and macro eco‐hopelessness. As modification indices indicated shared variance likely due to overlapping phrasing, residual covariances were included between corresponding items across the micro and macro subscales (e.g. Item 1 with Item 1, Item 2 with Item 2, etc.). These items were deliberately worded in parallel to assess the same underlying hopelessness in relation to different objects. Standardized factor loadings ranged from .43 to .88 for micro eco‐hopelessness and from .44 to .93 for macro eco‐hopelessness and were all statistically significant. The resulting model offered a theoretically coherent structure with good model fit and internal consistency for both subscales (*α*_Micro_ = .84; *α*_Macro_ = .79), Cronbach's alpha remained stable if items were dropped (*α*_Micro_ > .76; *α*_Macro_ > .70), and item‐rest correlations were within the acceptable range (*r*_*ir(Micro)*_ > .70; *r*_*ir(Micro)*_ > .43; the exact statistics can be found in Table S3). AVE indicated good convergent validity for both constructs, with values of .54 for *micro hopelessness* and .58 for *macro hopelessness*. Although HTMT ratio slightly exceeded the conservative cutoff of .85 (HTMT = .86), it remained below the more liberal threshold of .90 (Henseler et al., 2015). Given the theoretical distinction between the constructs and supporting evidence from the measurement model, discriminant validity was considered acceptable.

As in Study 1, well‐being was assessed via two components: emotional well‐being and life satisfaction, using the same validated instruments—the WHO‐5 Well‐Being Index and the SWLS (Diener et al., 1985). Both scales again demonstrated good internal consistency (*α*_WHO‐5_ = .88, *α*_SWLS_ = .81).

Similarly, the same five items as in Study 1 were used to measure PEB (see Table S2). Again, a CFA supported a one‐factor solution: *χ* ^2^(5) = 11.50, *p* = .04; CFI = .99; TLI = .98; RMSEA = .06; SRMR = .02. All factor loadings were statistically significant (*p* < .001) and ranged from .70 to .88. Reliability of the scale was good (*α* = .84).

#### Statistical analysis

In a first step, a multigroup SEM approach was used to examine whether the measurement of the variables and the structural relations among the latent constructs are comparable across the studies. Studies 1 and 2 served as the grouping variable. A sequence of increasingly constrained models was estimated. First, a baseline model was specified in which the same factor structure was imposed across groups, but all parameters were freely estimated. The estimated model corresponded exactly to the theory‐driven structural path model (M3) defined in Study 1. Specifically, eco‐worry and eco‐hopelessness were modeled as predictors of PEB and well‐being outcomes, respectively, while accounting for shared variance among latent constructs. Age and gender were included as control variables. Next, metric invariance was tested by constraining all factor loadings to equality across the two study samples. After establishing acceptable metric invariance, structural invariance was examined imposing constraints on the theory‐driven structural paths among the latent variables (i.e. the effects of eco‐worry and eco‐hopelessness on well‐being and PEB, respectively). The equality constraints were imposed on the unstandardized regression coefficients, yielding pooled estimates that were identical across studies. Given the sensitivity of *χ* ^2^ tests to sample size, nested models were compared using changes in fit indices (ΔCFI, ΔTLI, ΔRMSEA, and ΔSRMR) to assess whether additional constraints resulted in meaningful reductions in model fit. Comparing the freely estimated and constrained models enables a direct test of whether the hypothesized relationships are statistically equivalent across the two study samples. If constraining the structural paths does not result in a deterioration of the model fit, the relations can be interpreted as stable and replicated across the studies. Therefore, this multigroup SEM approach provides added value beyond single‐sample analyses by formally testing the robustness of a theoretically grounded structural model across independent samples and strengthens the generalizability of the findings despite the cross‐sectional nature of the data. Models were estimated with ML estimation, and data were handled using FIML.

In a second step, to further distinguish between the micro and macro dimensions of eco‐worry and eco‐hopelessness, the same stepwise modeling strategy involving three SEMs with increasing constraints as in Study 1 was applied. The latent covariance baseline model (M1) allowed all latent constructs to freely covary and served as an exploratory reference model. This model approximated the observed correlation matrix without imposing directional or structural assumptions while controlling for age and gender. The control variables were specified as predictors of all latent constructs and were allowed to covary with one another. Additionally, residual covariances were specified between eco‐hopelessness indicators that assessed similar item content across micro and macro levels (see CFA of eco‐hopelessness). The restricted covariance model (M2) introduced structural simplification by retaining only theoretically relevant covariances. This restricted covariance model functioned as an intermediate step between the latent covariance baseline model (M1) and the structural path model (M3). By reducing model complexity while preserving the most meaningful associations, M2 allows to assess whether a more parsimonious model could adequately capture the key structure of the data without compromising overall fit. Finally, the structural path model (M3) specified directional paths to test the study's hypotheses. The analyses employed ML estimation, with all constructs modeled as latent variables indicated by their respective items. FIML was used. All calculations were conducted using IBM SPSS Statistics (version 29.0.1.0), JASP (version 0.19.1.0), R (version 4.4.3), and RStudio (version 2016.01.0) using the packages lavaan (Rosseel et al., 2025), deplyr (Wickham et al., 2019), and semTools (Jorgensen et al., 2025). No missing data were observed for the variables under investigation.

### Results

The results of Study 2 are presented in two parts. The first part reports the results of the multigroup SEMs, and the second part analyzes the distinct associations of the micro and macro dimensions of eco‐worry and eco‐hopelessness with well‐being and PEB again using structural equation modeling. Descriptive statistics and bivariate correlations for all variables of Study 2 are presented in Table 3. The correlations provide an initial overview of the relationships among eco‐emotions, well‐being, and PEB. However, detailed interpretation is omitted, as hypothesis testing was conducted via the SEMs reported below. Data and analysis materials are available online (https://osf.io/xhv4b/overview?view_only=b0940e727b07487ca0f28af470dee677).

**TABLE 3:** Descriptive statistics and correlations of Study 2.

	*M* (*SD*)	Range	1	2	3	4	5	6	7	8	9
1. Eco‐worry	2.40 (0.85)	1.00–4.90	—								
2. Micro eco‐worry	2.44 (0.85)	1.00–5.00	.77***								
3. Macro eco‐worry	3.02 (1.00)	1.00–5.00	.84***	.70***							
4. Micro eco‐hopelessness	2.43 (1.27)	1.00–6.75	.27***	.30***	.20***						
5. Macro eco‐hopelessness	3.22 (1.37)	1.00–6.75	.34***	.31***	.27***	.70***					
6. Emotional well‐being	3.92 (0.89)	1.20–6.00	−.13*	−.16**	−.13*	−.34***	−.29***				
7. Life satisfaction	5.17 (1.08)	1.80–7.00	−.03	−.10	−.01	−.37***	−.17**	.53***			
8. PEB	3.00 (0.83)	1.00–5.00	.58***	.40***	.56***	.02	.05	.03	.08		
9. Age	34.93 (15.70)	18.00–84.00	−.02	−.13*	−.03	−.01	−.04	.19***	.12*	.11	
10. Gender	1.50 (0.50)	—	.14*	.15**	.20***	−.04	−.05	.04	.09	.12*	.17**

*Note*: Gender: 1 = male, 2 = female.

Abbreviations: PEB, pro‐environmental behavior; *SD*, standard deviation.

* *p* < .05,

** *p* < .01, and

*** *p* < .001.

#### Multigroup SEM

Multigroup structural equation modeling was conducted to examine whether the measurement and structural relations among the latent constructs were comparable across Studies 1 and 2. The baseline model showed acceptable fit to the data, *χ* ^2^(836) = 1779.67, CFI = .911, TLI = .900, RMSEA = .059, SRMR = .066, indicating that the hypothesized factor structure was similar across both studies. Next, metric invariance was tested by constraining all factor loadings to equality across the studies. This resulted in a statistically significant *χ* ^2^ difference test relative to the baseline model (Δ*χ* ^2^[24] = 58.25, *p* < .001). However, changes in fit indices were only small (ΔCFI = −.003, ΔTLI = −.001, ΔRMSEA = .000, ΔSRMR = .004). Thus, metric invariance was considered acceptable. Structural invariance was subsequently examined by constraining the theory‐driven regression paths among the latent variables to equality across groups. The resulting model continued to show acceptable fit (*χ* ^2^[863] = 1838.89, CFI = .907, TLI = .900, RMSEA = .059, SRMR = .070). Compared with the metric invariance model, constraining the structural paths (structural invariance model) did not significantly worsen model fit (Δ*χ* ^2^[3] = 0.98, *p* = .806) and was associated with only small changes in fit indices (ΔCFI = .000; ΔTLI = .001; ΔRMSEA = .000; ΔSRMR = .000). This indicates that the structural relations among the latent constructs were comparable across the two studies. The resulting standardized effects for the regression paths in Study 2 can be compared with the results of Study 1 in Figure 1.

Thus, the structural relations specified in the theory‐driven structural path model (M3) can be considered stable and replicated across Studies 1 and 2. Overall, the multigroup SEM results provide converging evidence that the theory‐driven structure underlying the structural path model (M3) introduced in Study 1 generalizes across both studies. The consistency of structural relations across samples strengthens confidence in the robustness of the findings despite the cross‐sectional design.

#### Structural equation model examining micro and macro dimensions

To analyze the distinct associations of the micro and macro dimensions of eco‐worry and eco‐hopelessness with well‐being and PEB, three sequential SEMs were estimated.

##### Latent covariance baseline model (M1)

The latent covariance model (M1) allowed all latent constructs to covary freely while controlling for age and gender. Model fit was acceptable (*χ* ^2^[457] = 860.98, *p* < .001, CFI = .94, RMSEA = .05, SRMR = .07, GFI = .97). See Table 4 for all covariances. Consistent with both the results of Study 1 and Hypothesis 1 micro and macro eco‐hopelessness were negatively associated with well‐being (emotional well‐being and life satisfaction). The associations indeed were stronger for micro eco‐hopelessness compared with macro eco‐hopelessness, which is in accordance with Hypothesis 1a. Contrary to Hypothesis 2, both dimensions of eco‐worry were negatively related to well‐being. However, the associations were only weak, and for micro eco‐worry and life satisfaction not significant, which questions a meaningful relationship between eco‐worry and individuals' overall well‐being. In line with Hypothesis 3, both micro‐ and macro‐level eco‐worry were positively associated with PEB, with the macro‐level dimension showing a stronger association, thereby supporting Hypothesis 3a. Both dimensions of eco‐hopelessness also showed a positive association with PEB, contradicting Hypothesis 4. However, the associations were only weak. In addition, consistent with the findings from Study 1, the model once again revealed a positive association between eco‐worry and eco‐hopelessness. Further, well‐being and PEB, like in Study 1, showed no association. Again, a positive association was observed between emotional well‐being and life satisfaction, reinforcing the internal consistency of the well‐being measures.

**TABLE 4:** Standardized associations of the latent covariance baseline model (M1) and restricted covariance model (M2) of Study 2.

Association	M1	M2
Emotional well‐being ~~ eco‐hopelessness (macro)	−.33*** (.06)	−.25*** (.06)
Emotional well‐being ~~ eco‐hopelessness (micro)	−.42*** (.06)	−.36*** (.05)
Life satisfaction ~~ eco‐hopelessness (macro)	−.22** (.06)	−.17** (.06)
Life satisfaction ~~ eco‐hopelessness (micro)	−.45*** (.05)	−.41*** (.05)
Emotional well‐being ~~ eco‐worry (macro)	−.17** (.06)	
Emotional well‐being ~~ eco‐worry (micro)	−.19** (.06)	
Life satisfaction ~~ eco‐worry (macro)	−.13* (.06)	
Life satisfaction ~~ eco‐worry (micro)	−.05 (.06)	
PEB ~~ emotional well‐being	−.01 (.06)	
PEB ~~ life satisfaction	.07 (.06)	
PEB ~~ eco‐hopelessness (macro)	.19** (.06)	
PEB ~~ eco‐hopelessness (micro)	.14* (.06)	
PEB ~~ eco‐worry (macro)	.59*** (.04)	.55*** (.04)
PEB ~~ eco‐worry (micro)	.44*** (.05)	.38*** (.05)
Life satisfaction ~~ emotional well‐being	.66*** (.04)	.66*** (.04)
Gender → eco‐worry (macro)	.22*** (.06)	.22*** (.06)
Gender → eco‐worry (micro)	.16** (.06)	.16** (.06)
Age → eco‐hopelessness (macro)	−.14* (.06)	−.13* (.06)
Age → eco‐worry (micro)	−.24*** (.06)	−.24*** (.06)
Age → emotional well‐being	.19** (.06)	.19** (.06)

*Note*: Covariances represent standardized associations between latent constructs. Values are shown as standardized coefficient (standard error). For clarity, within (.69–.76) and between (.20–.45) construct covariances of the two eco‐emotions (micro and macro dimensions of eco‐worry and eco‐hopelessness) are not displayed in this table. Age and gender were modeled as predictors of all latent variables in both models; only significant paths are shown. Gender was coded as 1 = male, 2 = female.

Abbreviation: PEB, pro‐environmental behavior.

* *p* < .05,

** *p* < .01, and

*** *p* < .001.

##### Restricted covariance model (M2)

The restricted covariance model (M2) retained only theoretically meaningful covariances from M1. The covariances between eco‐hopelessness and PEB, as well as between eco‐worry and well‐being, were omitted. Additionally, the covariances between PEB and each of the well‐being indicators were excluded. All excluded covariances were either statistically non‐significant, had *p*‐values greater than .001. Model fit remained comparable (*χ* ^2^[465] = 888.15, *p* < .001, CFI = .93, RMSEA = .05, SRMR = .08, GFI = .97). Although differences for information criteria were slightly better for M1 (ΔAIC = 11.17; ΔBIC = 71.20), M2 offers a more parsimonious structure, with fewer estimated parameters while maintaining an adequate fit to the data and was therefore considered the preferred model. All significant associations of M1 that are included in M2 remained significant. See Table 4 for all covariances.

##### Structural path model (M3)

**FIGURE 2:**
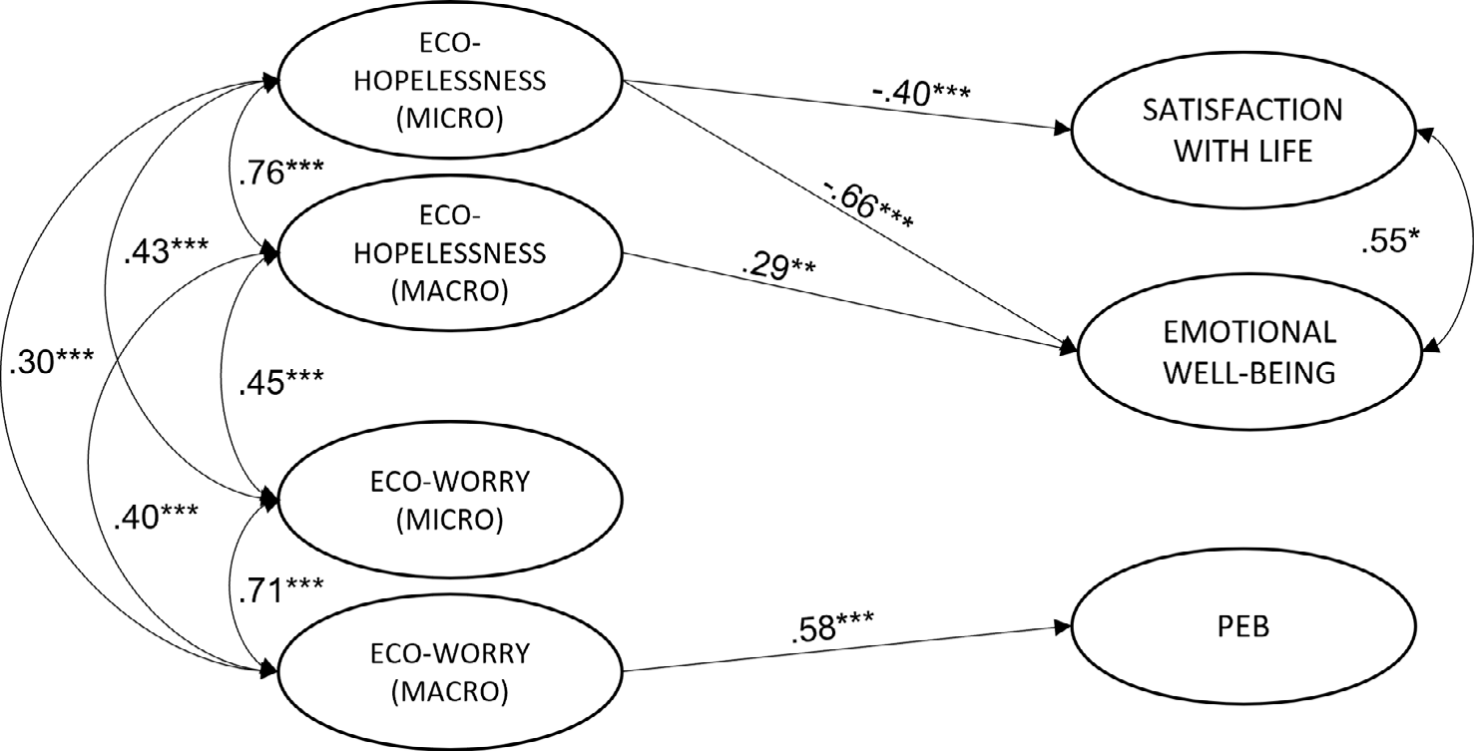
Structural path model (M3) of Study 2 showing the prediction of well‐being (emotional well‐being and life satisfaction) by micro and macro eco‐hopelessness and the prediction of PEB by micro and macro eco‐worries. Insignificant paths and paths for control variables (age, gender) are omitted for clarity. PEB, pro‐environmental behavior. *p < .05, **p < .01, and ***p < .001.

The structural path model (M3) specified directional paths based on the study's hypotheses. In particular, micro and macro eco‐hopelessness were modeled as predictors of emotional well‐being and life satisfaction, while micro and macro eco‐worries were modeled as predictors of PEB. Fit remained strong and on par with the previous models (*χ* ^2^[465] = 867.90, *p* < .001, CFI = .94, RMSEA = .05, SRMR = .07, GFI = .97). Therefore, M3 is considered the superior model, as it demonstrates solid model fit while only maintaining the hypothesized associations and examining theoretically supported directional effects. See Figure 2 for the results of M3.

As hypothesized (Hypothesis 1), eco‐hopelessness was negatively associated with well‐being. However, this was only true for micro eco‐hopelessness, which predicted both lower life satisfaction and lower emotional well‐being. Macro eco‐hopelessness, on the other hand, predicted higher emotional well‐being, contrary to Hypothesis 1, yet this association was considerably weaker. Consistent with the theoretical model, paths from eco‐worry to well‐being were not included, as no association was expected between these constructs. Because the overall model demonstrated good fit to the data, this provides support for Hypothesis 2, which proposed no relationship between eco‐worry and well‐being. Supporting Hypothesis 3, eco‐worry predicted higher PEB. As expected in Hypothesis 3a, this effect was driven by the macro‐level dimension, while micro‐level eco‐worry did not show a significant link. In line with the assumptions, paths from eco‐hopelessness to PEB were not specified, as no associations were hypothesized between these constructs. The comparable fit of M3 compared with M1 supports Hypothesis 4 that there is no link between eco‐hopelessness and PEB.

### Discussion

Overall, the results of Study 2 can be subsumed into parts. First, the multigroup structural equation modeling analyses revealed a consistent pattern of negative associations of eco‐hopelessness and well‐being outcomes across Study 1 and Study 2 while eco‐worry was not linked to well‐being, supporting Hypotheses 1 and 2. Therefore, perceiving environmental efforts as insufficient or feeling powerless in the face of climate challenges might play a critical role in an individual's psychological health. However, the structural path model (M3) revealed that micro‐level eco‐hopelessness showed a stronger and more consistent negative association with well‐being than macro eco‐hopelessness, which was only weakly positively related to emotional well‐being and not related to satisfaction with life, supporting Hypothesis 1a. This finding suggests that eco‐hopelessness related to the personal future is more closely linked to individuals' emotional and cognitive well‐being than feelings of eco‐hopelessness associated with the future of the world in general. Second, the multigroup structural equation modeling analyses further revealed a consistent relationship of eco‐worry with PEB across Study 1 and Study 2, indicating that higher levels of eco‐worry are related to greater engagement, which supports Hypothesis 3. In line with Hypothesis 4, eco‐hopelessness was not related to PEB, reinforcing the interpretation that eco‐hopelessness may not meaningfully motivate individual action in everyday life. When differentiating between micro and macro eco‐worries in the structural path model (M3), the positive association between eco‐worry and PEB holds true only for macro eco‐worry. For micro eco‐worry, no association was found, supporting Hypothesis 3a.

Overall, Study 2 both replicates and extends the findings of Study 1, with its central contribution being the additional differentiation between micro and macro dimensions of eco‐worry and eco‐hopelessness. Importantly, the results of Study 2 go beyond a mere repetition of effects. Evidence for measurement invariance from the multigroup SEMs indicates that the key constructs function equivalently across Study 1 and Study 2, while structural invariance demonstrates that the pattern and strength of associations among eco‐worry, eco‐hopelessness, PEB, and well‐being are stable across samples. Together, these findings provide a strong empirical basis for integrating the results of both studies. In addition, Study 2 highlights the importance of considering an individual versus a collective focus when examining psychological and behavioral correlates of eco‐worry and eco‐hopelessness. Although these dimensions were highly interrelated within each construct, their associations with well‐being and PEB differed in theoretically meaningful ways, providing additional support for the predictive validity of the measures.

## GENERAL DISCUSSION

The present research aimed to examine how two emotional responses to climate change—eco‐worry and eco‐hopelessness—relate to psychological well‐being and PEB. By conducting two studies and employing (multigroup) SEM, I sought to clarify whether eco‐worry and eco‐hopelessness are differentially associated with well‐being and PEB and whether distinctions between individual (micro) and collective (macro) perspectives further nuance these relationships.

Across both studies, eco‐worry and eco‐hopelessness emerged as moderately correlated constructs, where more worry is linked to greater feelings of hopelessness. However, their associations with well‐being and PEB differed in meaningful ways. As expected, eco‐hopelessness was consistently linked to lower well‐being in terms of emotional well‐being and life satisfaction across both studies, with Study 2 replicating the results of Study 1. This aligns with broader psychological literature identifying hopelessness as a critical risk factor for psychological impairment (Liu et al., 2015; Marchetti et al., 2023). Importantly, the findings extend this evidence to the environmental domain by demonstrating that climate‐specific hopelessness is also associated with lower well‐being. In contrast, eco‐worry showed no associations with the two components of well‐being in the theory‐driven SEMs. These findings suggest that eco‐worry does not inherently indicate impairment in well‐being. Rather, as suggested in previous research (e.g. Ojala et al., 2021), it may represent an adaptive emotional response motivating individuals to engage with climate issues without necessarily undermining their mental health, which is also in line with the appraisal theory of emotion (Smith & Kirby, 2011; Smith & Lazarus, 1990) because the outcome is uncertain and change might be possible. This distinction is particularly important given the growing tendency to pathologize eco‐worry in public discourse. Eco‐worry was consistently positively associated with PEB, supporting the notion that worry can serve as a motivational force (Bouman et al., 2020; Verplanken et al., 2020). Eco‐hopelessness, however, demonstrated no (or only weak) associations with PEB in both studies, contradicting assumptions that it may inhibit PEB due to its core feature of blocked goal‐pursuit (see Marchetti et al., 2023). These results suggest that although eco‐hopelessness is linked to impaired well‐being, its role in PEB may be less straightforward. It is possible that individuals can feel hopeless regarding the threat of climate change yet still act environmentally friendly, driven by, for example, social norms or moral obligation—an area warranting further research.

A novel contribution of this research lies in the distinction between micro‐ and macro‐dimensions of both eco‐worry and eco‐hopelessness. This perspective provided additional explanatory value, particularly regarding the associations with well‐being. In both studies, micro eco‐hopelessness (i.e. a pessimistic view of one's own future due to climate change) was more strongly associated with impaired well‐being than macro eco‐hopelessness (i.e. a pessimistic view of the future for society or the world). This aligns with earlier findings on the distinction between micro and macro worry in other domains (Boehnke et al., 1998) and extends these findings to a related but distinct psychological construct—eco‐hopelessness. This suggests that appraisals of climate change threat as certain and inevitable for one's own life might be more harmful than for the world at large. In contrast, macro‐eco‐worry, reflecting a broader worry for the planet or future generations, was more strongly associated with PEB than micro‐eco‐worry. This pattern is consistent with theories of value orientation (Schultz, 2001), where biospheric and altruistic concerns are more predictive of environmental engagement than egoistic values. Thus, the object of one's worries or despair matters, not just the presence of these emotions. These results can also be linked to research on the psychological distance of climate change. For example, Spence et al. (2012) found that closer psychological distance was related to levels of concern, and greater psychological distance was related to more PEB.

Finally, in both studies, well‐being indicators were not related to PEB. Although this was not a part of the primary research questions, I believe the results to be important. The lack of association in both my studies suggests that psychological well‐being may not directly drive PEB or that PEB does not lead to better well‐being. This finding underscores the importance of not assuming that promoting mental health will automatically translate into climate action, or vice versa. However, as Caldaroni et al. (2025) emphasize, one has to distinguish between hedonic (as measured in this research) and eudemonic well‐being. While PEB was not related to hedonic well‐being in my studies, Caldaroni et al. (2025) found that fostering PEB actually led to higher levels of eudemonic well‐being (here meaning in life). Future studies should investigate whether these distinct patterns of hedonic and eudemonic well‐being concerning PEB are consistent.

## LIMITATIONS

Although these studies provide valuable novel insights on the relationships of eco‐worry and eco‐hopelessness with well‐being and PEB, several limitations should be noted. First, although I provide replication of the findings, the cross‐sectional design precludes any causal conclusions. Future longitudinal or experimental designs are needed to clarify the directionality of the observed associations. Second, the reliance on self‐report measures, particularly for PEB, may have introduced social desirability bias. Third, although this study differentiated between micro and macro perspectives, previous research has proposed a more nuanced categorization of concern regarding climate change, resulting in a total of three objects of concern: egoistic, social, and biospheric (Helm et al., 2018; Schultz, 2001). Future research should investigate whether social and biospheric eco‐worry differ in their associations with well‐being and PEB, as the study by Helm et al. (2018) suggests. Fourth, additional research is necessary to explore how individuals use coping strategies to manage climate‐related emotions and how these strategies influence well‐being and behavior. This might be especially relevant since Wullenkord and Ojala (2023) argue that if worry is not efficiently coped with, it could lead to feelings of hopelessness. Coping strategies might also be able to buffer the negative relationship between eco‐hopelessness and well‐being.

Despite these limitations, this research contributes novel insights to environmental psychology by distinguishing between negative emotional responses to climate change and their psychological and behavioral correlates. The findings underscore the importance of moving beyond broad constructs like eco‐anxiety to more concrete emotions, such as eco‐worry and eco‐hopelessness, while also accounting for the object of these emotions.

## CONCLUSION

This research highlights that not all negative climate emotions function alike. Eco‐worry may motivate engagement without impairing well‐being, while eco‐hopelessness signals risk for psychological health. Moreover, the distinction between individual and collective perspectives of these emotions is important as the object of one's worry or hopelessness shows different relations to well‐being and PEB. The findings suggest that when eco‐worry or eco‐hopelessness are focused on personal impacts, they are more strongly associated with reduced well‐being. In contrast, a focus towards broader societal or global outcomes is more likely to be linked with increased PEB. This highlights the importance of not only examining the emotional responses themselves but also understanding the focus of these emotions. Interventions aiming to support mental health and promote climate action must therefore differentiate between emotional profiles and tailor strategies accordingly—empowering global worry while mitigating personal despair.

## CONFLICT OF INTEREST STATEMENT

The authors report there are no competing interests to declare.

## ETHICS STATEMENT

The studies were approved by the institutional Ethics Committee of the University of the Bundeswehr Munich.

## AI STATEMENT

OpenAI ChatGPT (GPT‐4.2–GPT‐5.2; accessed May 2025–January 2026) was used to (a) suggest code patterns during analysis scripting, (b) support literature search and organization, and (c) edit English phrasing for clarity and grammar. All model outputs were critically reviewed, revised, and validated by the author; references were checked against primary sources. No generative AI was used to create or manipulate original research data.

## Supporting information


**Table S1.** Items for micro and macro eco‐hopelessness.
**Table S2.** Items for pro‐environmental behavior (PEB).
**Table S3.** Items for micro and macro eco‐worry.

## Data Availability

The data that support the findings of this study are openly available in OSF (https://osf.io/xhv4b/overview?view_only=45d5b8a4160a466ca51261242d079a8d).
