# Supplementary material for: Motivated by worry, burdened by hopelessness: Emotional correlates of climate action and personal well‐being
Source: Appl Psychol Health Well Being. 2026 Jul 1;18(4):e70183. doi: 10.1111/aphw.70183 (PMC13322174; doi:10.1111/aphw.70183)
Supplement: Supplementary file 1 — Table S1. Items for micro and macro eco‐hopelessness. Table S2. Items for pro‐environmental behavior (PEB). Table S3. Items for micro and macro eco‐worry. [file APHW-18-0-s001.docx]

# Supplementary Material

**Table S1**

| *Items for micro and macro eco-hopelessness* | | | |
| --- | --- | --- | --- |
| Item | The following questions relate to your attitude toward climate change in relation to your personal future (micro) / the world and society in general (macro): | Cronbach’s α if item dropped | Item-rest correlation |
| Micro 01 | I have the feeling that my future is hopeless and that things cannot improve | .76 | .72 |
| Micro 02 | My future seems dark to me | .77 | .73 |
| Micro 03 | Things just won't work out the way I want | .78 | .70 |
| Micro 04 | There is no point I really try to change anything because it probably won't work | .87 | .50 |
| Macro 01 | I have the feeling that the future is hopeless and that things cannot improve | .70 | .69 |
| Macro 02 | The future seems dark to me | .71 | .68 |
| Macro 03 | Things just won't work out the way one wants | .73 | .63 |
| Macro 04 | There is no point in really trying to change anything because it probably won't work | .82 | .43 |

| **Table S2** | |
| --- | --- |
| *Items for pro-environmental behavior (PEB)* | |
| 1 | I behave sustainably. |
| 2 | I reduce behaviors that contribute to climate change. |
| 3 | I take action to address the problem of climate change. |
| 4 | I make sacrifices in my consumption habits to combat climate change. |
| 5 | I try to convince others to adopt climate-neutral behavior. |
|  | |

**Table S3**

| *Items for micro and macro eco-worry* | | | |
| --- | --- | --- | --- |
| Item | I am concerned about environmental problems because of the consequences for… | Cronbach’s α if item dropped | Item-rest correlation |
| Micro 01 | …my lifestyle | .89 | .55 |
| Micro 02 | … my future | .79 | .81 |
| Micro 03 | … my health | .79 | .79 |
| Micro 04 | … me | .83 | .71 |
| Micro 05 | … my family/friends | excluded |  |
| Macro 01 | … the society | .90 | .81 |
| Macro 02 | … all people | .88 | .87 |
| Macro 03 | … future generations | .91 | .78 |
| Macro 04 | … the world | .90 | .78 |
| Macro 05 | … nature and/or animals | excluded |  |
